# Left Ventricular End-Systolic Volume Is a Reliable Predictor of New-Onset Heart Failure with Preserved Left Ventricular Ejection Fraction

**DOI:** 10.1155/2020/3106012

**Published:** 2020-06-29

**Authors:** Marina Kato, Shuichi Kitada, Yu Kawada, Kosuke Nakasuka, Shohei Kikuchi, Yoshihiro Seo, Nobuyuki Ohte

**Affiliations:** Department of Cardiology, Nagoya City University, Nagoya, Japan

## Abstract

**Background:**

Left ventricular (LV) ejection fraction (EF) and LV volumes were reported to have prognostic efficacy in cardiac diseases. In particular, the end-systolic volume index (LVESVI) has been featured as the most reliable prognostic indicator. However, such efficacy in patients with LVEF ≥ 50% has not been elucidated.

**Methods:**

We screened the patients who received cardiac catheterization to evaluate coronary artery disease concomitantly with both left ventriculography and LV pressure recording using a catheter-tipped micromanometer and finally enrolled 355 patients with LVEF ≥ 50% and no history of heart failure (HF) after exclusion of the patients with severe coronary artery stenosis requiring early revascularization. Cardiovascular death or hospitalization for HF was defined as adverse events. The prognostic value of LVESVI was investigated using a Cox proportional hazards model.

**Results:**

A univariable analysis demonstrated that age, log BNP level, tau, peak − d*P*/d*t*, LVEF, LV end-diastolic volume index (LVEDVI), and LVESVI were associated with adverse events. A correlation analysis revealed that LVESVI was significantly associated with log BNP level (*r* = 0.356, *p* < 0.001), +d*P*/d*t* (*r* = −0.324, *p* < 0.001), −d*P*/d*t* (*r* = 0.391, *p* < 0.001), and tau (*r* = 0.337, *p* < 0.001). Multivariable analysis with a stepwise procedure using the variables with statistical significance in the univariable analysis revealed that aging, an increase in BNP level, and enlargement of LVESVI were significant prognostic indicators (age: HR: 1.071, 95% CI: 1.009–1.137, *p*=0.024; log BNP : HR : 1.533, 95% CI: 1.090–2.156, *p*=0.014; LVESVI : HR : 1.051, 95% CI: 1.011–1.093, *p*=0.013, respectively). According to the receiver-operating characteristic curve analysis for adverse events, log BNP level of 3.23 pg/ml (BNP level: 25.3 pg/ml) and an LVESVI of 24.1 ml/m^2^ were optimal cutoff values (BNP : AUC : 0.753, *p* < 0.001, LVESVI : AUC : 0.729, *p* < 0.001, respectively).

**Conclusion:**

In patients with LVEF ≥ 50%, an increased LVESVI is related to the adverse events. LV contractile performance even in the range of preserved LVEF should be considered as a role of a prognostic indicator.

## 1. Introduction

Left ventricular (LV) ejection fraction (EF) and LV end-diastolic and end-systolic volumes (LVEDV and LVESV, respectively) are commonly used as clinical parameters reflecting global LV systolic performance or LV remodeling [1, 2]. Of note, compared with LVEF or LVEDV, previous reports emphasized the superiority of LVESV (or LVESV indexed to the area of body surface: LVESVI) in predicting poor prognosis in patients with cardiac disease [3–6]. According to the latest reports concerned with the prognosis of patients who underwent surgical treatment for severe mitral regurgitation due to mitral valve prolapse, LV end-systolic diameter still maintained the position as a prognostic indicator [7, 8]. In addition, the prognostic utility of LVESVI in patients with stable coronary artery disease was also reported to be superior to that of LVEF or LVEDV index (LVEDVI) [5]. Gilbert and Glantz [9] previously showed that a relatively smaller LV chamber in end-systole, which stored elastic energy during systole, could produce a greater degree of LV recoil force during the isovolumic relaxation and resulted in better LV relaxation. Therefore, we hypothesized that even in patients with LVEF ≥ 50%, slightly impaired LV systolic function, which is sensitively reflected in an increase in LVESVI, and subsequent prolonged LV relaxation were common mechanisms associated with cardiac death and heart failure (HF). Accordingly, we investigated the prognostic power of LVESVI for adverse events in patients with LVEF ≥ 50%.

## 2. Methods

### 2.1. Study Population

In consecutive 465 patients who underwent cardiac catheterization to diagnose possible coronary artery disease and comprehensive cardiac function analysis from May 1999 to January 2011, LVESV as well as LVEDV was measured using biplane contrast left ventriculography and LV pressure was recorded using a catheter-tipped micromanometer. In this cohort, we retrospectively enrolled a total of 355 patients who satisfied the eligible inclusion and exclusion criteria of this study. The inclusion criteria consisted of age, 20 years or older; LVEF ≥ 50%; no history of hospitalization for HF prior to the enrollment; and no change in baseline drug therapy for 1 month prior to the enrollment. The exclusion criteria were acute coronary syndrome requiring urgent coronary revascularization and severe coronary artery stenosis with symptoms suggestive of myocardial ischemia or coronary stenosis from 50 to 90% with myocardial ischemia. The myocardial ischemia was diagnosed by exercise or drug-induced stress electrocardiogram, myocardial nuclear perfusion imaging, or echocardiography; serum creatinine level > 2.5 mg/dL; old myocardial infarction resulting in LVEF < 50%; hypertrophic cardiomyopathy or infiltrative cardiomyopathy such as amyloid cardiomyopathy; cardiac rhythm other than sinus rhythm including pacemaker rhythm; hemodynamically significant aortic or mitral valve disease; and any serious noncardiovascular disease expected to live within 6 months such as malignancy.

### 2.2. Data Collection

We collected data on demographics, laboratory values, medication, LV volumes, and LV function parameters. Before using contrast agents in cardiac catheterization, we first computed cardiac output (CO) with a thermodilution method and calculated the cardiac index (CI) where CO was normalized by the body surface area. We then obtained LV pressure using a catheter-tipped micromanometer (SPC-454D, Millar Instrument Co., Houston, Texas) and it was recorded on a polygraph system (RMC-2000 or RMC-3000, Nihon Kohden Inc., Tokyo, Japan) and also on a digital data recorder (NR-2000, Keyence, Osaka, Japan) at a sampling interval of 2 ms. From the LV pressure recording, we determined peak positive and negative first derivatives of LV pressure (peak ± *d*P/*d*t) and computed a time constant tau of LV pressure decay during isovolumic relaxation using the method proposed by Weiss et al. [10] After all pressure recording, left ventriculography and coronary angiography were performed. On left ventriculography, LVESV and LVEDV were measured using the method described by Chapman et al. [11]. Then, LVEF was determined. Both LVESV and LVEDV were normalized by the body surface area of each patient and expressed as LVEDVI and LVESVI. The study endpoint was defined as a composite of cardiovascular death or unplanned hospitalization due to acute decompensated HF. Outcome data of study patients were also collected retrospectively.

### 2.3. Statistical Analysis

Continuous data are presented as the mean ± SD, and categorical variables are summarized as frequency and percentage. We evaluated the contribution of each clinical variable to the relative hazard of experiencing the composite endpoint of this study using a Cox proportional hazards model. Then, we assessed the correlations of LVESVI with the variables which reached statistical significance in the univariable analysis. Furthermore, we evaluated the contribution of clinical variables using a multivariable Cox regression analysis with a stepwise procedure using the variables with a statistical significance in the univariable analysis. We defined the day of cardiac catheterization as the time of enrollment of a patient in this study. And we adopted the duration of observation as the time from the enrollment to the occurrence of a terminal endpoint or the last censoring when patients were survived without adverse events during the follow-up period. Besides, when the study patients needed percutaneous coronary revascularization or surgical coronary artery bypass grafting during their follow-up period, these patients were defined as censored cases and the duration between the enrollment and the time of coronary interventions was adopted as an observation period for them. A *p* value <0.05 was considered statistically significant. The optimal cutoff value of clinical variables with statistical significance in the multivariable analysis was also assessed using a receiver-operating characteristic (ROC) curve analysis for the composite endpoint of this study. Then, the whole study patients were divided into 2 groups using the optimal cutoff values. The Kaplan-Meier event-free survival curves in these 2 groups were compared by the log-rank test.

All statistical analyses were performed with SPSS version 23.0 software (SPSS Japan Inc., Tokyo). This study was conducted in full accordance with the Declaration of Helsinki, and it received approval from the Institutional Review Boards and Ethics Committees of the Nagoya City University Graduate School of Medical Sciences.

## 3. Results

### 3.1. Clinical Characteristics of Patients

The clinical characteristics of the whole patients are shown in Table 1. The mean age of patients was 67.4 years and 90 patients (25.4%) were female. The patients who had a past history of myocardial infarction were 38.0%. More than half of the patients had either hypertension (58.6%) or hyperlipidemia (58.6%), or both. Additionally, 36.3% of patients had diabetes mellitus as comorbidity. The mean value of LVEF was 68.7% and the median BNP level was in the normal range (15.6 pg/ml; interquartile range (IQR): 8.1 and 36.3 pg/ml).

### 3.2. Contribution of Cardiac Function Parameters to the Prognosis and Associations of LVESVI with the Parameters Which Showed Prognostic Power to Predict the Adverse Events

During the follow-up period (median: 2430 days; IQR: 1480 and 3332 days), 9 cardiovascular deaths and 15 hospitalizations for HF were documented. Among 355 patients who were enrolled in this study, 41 patients needed percutaneous coronary revascularization and 2 patients underwent surgical coronary artery bypass grafting during their follow-up period. All of them did not experience concomitant HF.

**Figure 1:**
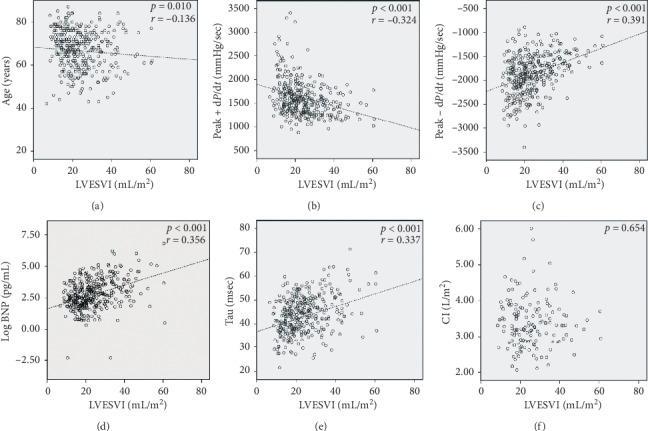
Associations of LVESVI with age, BNP levels, sophisticated cardiac performance parameters such as tau, peak ± d*P*/d*t*, and CI. LVESVI was significantly correlated with BNP levels, peak ± d*P*/d*t*, tau, and age; however, it showed no correlation with CI.

In Table 2, the contribution of each parameter to the composite endpoint was demonstrated. In univariable analyses, age, log BNP level, tau, peak − d*P*/d*t*, LVEF, LVEDVI, and LVESVI showed significant associations with adverse events. The correlation analyses were performed to examine the associations of LVESVI with these parameters and CI (Figure 1). We found that the LVESVI was significantly correlated with log BNP levels (*r* = 0.356, *p* < 0.001) as well as the peak ± d*P*/d*t*, and tau (peak + d*P*/d*t*, *r* = −0.324, *p* < 0.001; peak − d*P*/d*t*, *r* = 0.391, *p* < 0.001; tau, *r* = 0.337, *p* < 0.001, respectively).

In the multivariable Cox proportional hazards model which used all parameters with statistical significance in the univariable analysis, aging, an increase in BNP level, and enlargement of LVESVI were selected as significant predictors of the adverse events (age, HR: 1.071, 95% CI: 1.009 to 1.137, *p*=0.024; log BNP, HR: 1.533, 95% CI: 1.090 to 2.156, *p*=0.014; LVESVI, HR: 1.051, 95% CI: 1.011 to 1.093, *p*=0.013, respectively).

### 3.3. Optimal Cutoff Values of Prognostic Indicators for the Adverse Events

**Figure 2:**
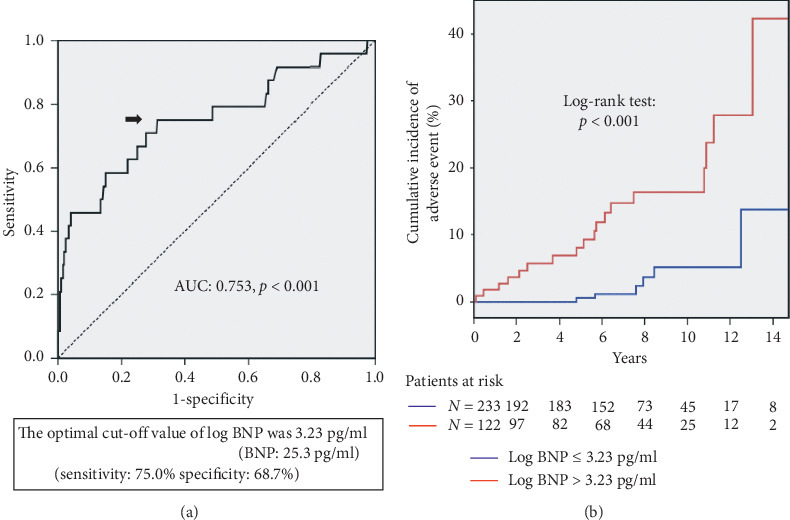
ROC curve of log BNP level for cardiovascular death or hospitalization for new-onset heart failure in patients with preserved LVEF. An optimal cutoff value of log BNP level was indicated by an arrow. Log BNP level of 3.23 pg/ml (BNP level was 25.3 pg/ml) showed 75.0% sensitivity and 68.7% specificity (AUC: 0.753, *p* < 0.001). The comparison of the event-free survival curves revealed that the patients with log BNP level > 3.23 pg/ml (in red) had a worse event-free survival compared to those with log BNP level ≤ 3.23 pg/ml (in blue) (log-rank test, *p* < 0.001).

**Figure 3:**
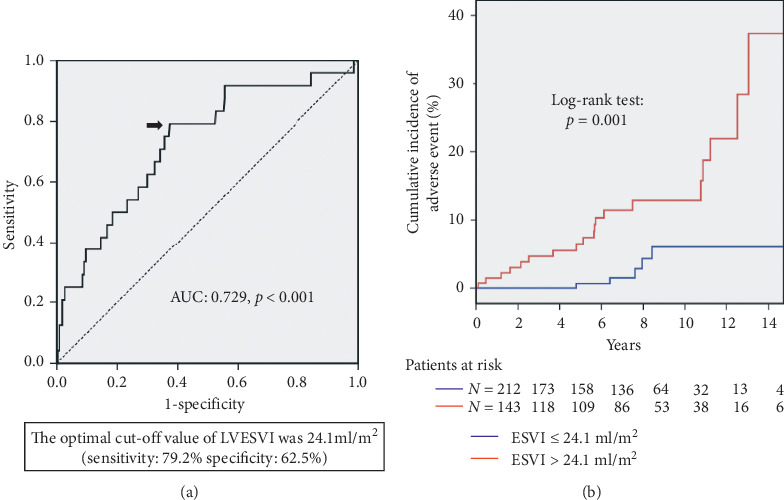
ROC curve of LVESVI for cardiovascular death or hospitalization for new-onset heart failure in patients with preserved LVEF. An optimal cutoff value of LVESVI was indicated by an arrow. LVESVI of 24.1 ml/m^2^ showed 79.2% sensitivity and 62.5% specificity (AUC: 0.729, *p* < 0.001). The comparison of the event-free survival curves revealed that the patients with LVESVI > 24.1 ml/m^2^ (in red) had a worse event-free survival compared to those with LVESVI ≤ 24.1 ml/m^2^ (in blue) (log-rank test, *p*=0.001).

According to the ROC curve analysis to predict the composite endpoint in this study (Figure 2(a)), the optimal cutoff value of log BNP level was 3.23 pg/ml (BNP level: 25.3 pg/ml) with a sensitivity of 75.0% and a specificity of 68.7% (AUC: 0.753, *p* < 0.001). Compared to the patients with log BNP level ≤3.23 pg/ml, those with log BNP level >3.23 pg/ml showed significantly worse event-free survival (log-rank test: *p* < 0.001) (Figure 2(b)). In addition, the optimal cutoff value of LVESVI was 24.1 ml/m^2^ with a sensitivity of 79.2% and a specificity of 62.5% (AUC: 0.729, *p* < 0.001) (Figure 3(a)). Compared to the patients with LVESVI ≤ 24.1 ml/m^2^, those with LVESVI > 24.1 ml/m^2^ showed a significantly worse event-free survival (log-rank test: *p*=0.001) (Figure 3(b)).

## 4. Discussion

In the current study, we demonstrated that an enlargement of LVESVI as well as an increase in BNP level and aging had a significant power to predict cardiac death and de novo HF even in patients with LVEF ≥50%. In contrast, LVEF and LVEDVI were not selected as an independent parameter related to the outcome in the multivariable analysis. BNP and N-terminal proBNP (NT-proBNP) are described in the current guidelines as gold standard biomarkers for the diagnosis and evaluation of the prognosis of HF. Furthermore, natriuretic peptide level-guided risk stratification of patients with symptoms suggestive HF is used in clinical practice [12, 13]. Consistently, in our study, a slight increase in BNP level (>25.3 pg/ml) was associated with adverse events including new-onset HF in patients with LVEF ≥ 50%. However, the usefulness of the LV volume parameter such as LVESVI for future risk stratification has not become widely acknowledged. Our findings may highlight the role of LVESVI in relation to the future occurrence of the adverse events in patients with LVEF > 50%. Even in patients with LVEF > 50%, LV contractile performance has an impact on the patient's prognosis.

In patients with HF, LVEF and LV volumes are reflecting global LV systolic performance or associated with LV remodeling [1, 2]. Coinstantaneously, considerable compensatory change of LVEF and LV volumes may be accompanied by HF. Therefore, LVEF, LVEDV, and LVESV have a possibility of tightly reflecting the status of patients with HF and of associating with their morbidity and mortality [14, 15]. In particular, the superiority of LVESV compared with LVEF and LVEDV in the predictive value for poor prognosis in patients with cardiac disease has been featured in several previous reports. One report demonstrated that LVESV had a greater predictive value for survival in the patients recovered from myocardial infarction than LVEDV or LVEF [4]. Additionally, in patients with stable coronary heart disease, the comparison of area under the ROC curves for the prediction of future hospitalization for HF demonstrated that LVESVI was superior to LVEDVI and LVEF as a predictor [5]. Furthermore, for children with cardiac disease and adult patients with valvular regurgitation, such a strong prognostic power of LVESV was also demonstrated [3–6]. Consistently, in the current study, we demonstrated that an increase in LVESVI was a risk for the adverse events in patients with LVEF ≥ 50%, while LVEF did not.

The reliability of LVESV as a prognostic indicator may arise from the higher sensitivity of LVESV for the ventricular contractile performance change. LVESV is determined directly by LV contractility as well as afterload to the left ventricle and not dependent on LVEDV. LVEDV is determined by the mechanism presented by Frank and Starling according to the given LVESV [16]. Hence, as LV reduces its contractility, LVESV is enlarged while the afterload remains constant. Recently, some reports using echocardiography have shown that HF with preserved EF (HFpEF) was associated with slight impairment of LV contractility [17, 18]. According to the study by Shah et al. [19], an abnormality in LV contractility could be detected by a reduction in the longitudinal strain of the left ventricle. The reduction of LV strain had prognostic value in patients with HFpEF in the TOPCAT trial (Treatment of Preserved Cardiac Function Heart Failure with an Aldosterone Antagonist) [20]. LVESV can be obtained without the use of such new technology as myocardial strain imaging.

Most patients with HFpEF showed normal LV filling pressure at rest; however, an abnormality in LV filling pressure developed during physiological stress like exercise or volume loading [21–23]. Compared to LVEF, LVESV demonstrated higher sensitivity for the ventricular contractile reserve to the same physiologic increases in preload and afterload. Turakhia et al. [24] demonstrated that an increase in LVESV during exercise treadmill testing, which was evaluated as the difference between the LVESV before exercise and that during exercise, was associated with exercise intolerance and independently predicted mortality in patients with stable coronary heart disease and normal LVEF (LVEF ≥ 55%). An increase in LVESV may be more sensitive than a decrease in LVEF for the detection of the impairment of LV contractility.

A small LVESV indicated that LV maintained not only good contractility but also a strong elastic recoil force of myocardium. Thus, it has been used as a parameter of elastic recoil of the left ventricle [9, 25]. During systole, the contractile elements of the left ventricle decrease the length of the muscle fibers. When the myocardial fiber length decreases below its resting length, left ventricles start storing potential energy. Then, the left ventricles continue to store it until the time of end-systole. Finally, this energy is released during early diastole and produces active suction of blood from the left atrium, contributing to rapid LV filling, resulting in a good LV pump performance. We previously demonstrated the strong association between a decrease in LV contractile performance and new-onset HF in patients with preserved LVEF [26, 27]. The contractile performance parameter showed strong correlations with tau and peak − d*P*/d*t*, suggesting that strong elastic recoil force existed concurrently with good contractile performance in the left ventricle [9]. Similarly, the current study demonstrated that LVESVI had significant positive correlations with tau and peak − d*P*/d*t*. These findings suggested that an increase in LVESVI concomitant with impairment of LV relaxation could be a prognostic indicator of new-onset HF.

This study had several limitations. First, we analyzed data retrospectively in a single institution. Second, the study patients were with a low incidence of new-onset HF. Most patients had some risk factors for HFpEF, such as hypertension, diabetes mellitus, and coexistence of coronary arteriosclerosis not requiring early revascularization. In addition, the median BNP level was in the normal range and all patients were in sinus rhythm. Thus, a future prospective study is needed to strengthen our conclusions, with a larger study cohort that includes more patients with a history of hospitalization for HFpEF.

In conclusion, in addition to an increase in BNP level and aging, an enlargement of LVESVI has significant prognostic power for adverse events even in patients with LVEF ≥ 50%. An enlargement of LVESVI exceeding 24.1 ml/m^2^ is associated with the future occurrence of the adverse events in such patients. Even in patients with LVEF >50%, LV contractile performance does have an impact on the patient's prognosis.

## Figures and Tables

**Table 1:** Patient characteristics.

Whole cohort (*n* = 355)	
Age, years	67.4 ± 9.3
Female	90 (25.4)
BSA (m^2^)	1.67 ± 0.18
Systolic BP (mmHg)	128 ± 18
Heart rate (beats/min)	67 ± 12

Laboratory data	
Hemoglobin (g/dl)	13.4 ± 1.5
Creatine (mg/dl)	0.83 ± 0.18
BNP, pg/ml (IQR)	15.6 (8.1, 36.3)
log BNP (pg/ml)	2.81 ± 1.17

Cardiac function parameter	
Tau (msec)	44.0 ± 7.8
peak + d*P*/d*t* (mmHg/sec)	1599.8 ± 374.6
peak − d*P*/d*t* (mmHg/sec)	−1869.0 ± 406.4
CI (l/min/m^2^)	3.39 ± 0.68
LVEF (%)	68.7 ± 8.7
LVEDVI (ml/m^2^)	74.7 ± 16.6
LVESVI (ml/m^2^)	23.8 ± 10.1
LVEDP (mmHg)	14 ± 5

Comorbidities, *n* (%)	
Hypertension	208 (58.6)
Diabetes	129 (36.3)
Hyperlipidemia	208 (58.6)
Past history of MI	135 (38.0)

Medication, *n* (%)	
ACEI and/or ARB	141 (39.7)
Beta blocker	118 (33.2)
CCB	105 (29.6)
Diuretic agent	21 (5.9)

ACEI, angiotensin converting enzyme inhibitor; ARB, angiotensin receptor blocker; BP, blood pressure; BNP, brain natriuretic peptide; BSA, body surface area; CCB, calcium channel blocker; CI, cardiac index; d*P*/d*t*, the first derivative of left ventricular pressure; LVEF, left ventricular ejection fraction; LVEDP, left ventricular end-diastolic pressure; LVEDVI, left ventricular end-diastolic volume index; LVESVI, left ventricular end-systolic volume index; MI, myocardial infarction.

**Table 2:** Results of multivariable Cox proportional hazards regression model analysis.

	Univariable		Multivariable	
	HR (95% CI)	*p* value	HR (95% CI)	*p* value
Age, years	1.093 (1.032–1.157)	0.002	1.071 (1.009–1.137)	0.024
Female		0.629		
BSA (m^2^)		0.315		
Systolic BP (mmHg)		0.841		
Heart rate (beats/min)		0.435		
Hemoglobin (g/dl)		0.482		
Creatine (mg/dl)		0.560		
Log BNP (pg/ml)	**2.120 (1.542–2.915)**	**<0.001**	**1.533 (1.090–2.156)**	**0.014**
Tau (msec)	**1.061 (1.009–1.114)**	**0.020**		0.205
Peak + d*P*/d*t* (mmHg/sec)		0.210		
Peak − d*P*/d*t* (mmHg/sec)	**1.001 (1.000–1.002)**	**0.019**		0.496
CI (l/min/m^2^)		0.692		
LVEF (%)	**0.935 (0.891–0.982)**	**0.007**		0.249
LVEDVI (ml/m^2^)	**1.035 (1.013–1.058)**	**0.002**		0.351
LVESVI (ml/m^2^)	**1.059 (1.027–1.092)**	**<0.001**	**1.051 (1.011–1.093)**	**0.013**
LVEDP (mmHg)		0.084		
Hypertension		0.305		
Diabetes		0.356		
Hyperlipidemia		0.874		
Past history of MI		0.388		
ACEI and/or ARB		0.456		
Beta blocker		0.389		
CCB		0.427		
Diuretic agent		0.856		

ACEI, angiotensin converting enzyme inhibitor; ARB, angiotensin receptor blocker; BP, blood pressure; BNP, brain natriuretic peptide; BSA, body surface area; CCB, calcium channel blocker; CI, cardiac index; d*P*/d*t*, the first derivative of left ventricular pressure; LVEF, left ventricular ejection fraction; LVEDP, left ventricular end-diastolic pressure; LVEDVI, left ventricular end-diastolic volume index; LVESVI, left ventricular end-systolic volume index; MI, myocardial infarction.

## Data Availability

The data used to support this study are available upon request to the corresponding author.
